# Intraorbital findings in giant cell arteritis on black blood MRI

**DOI:** 10.1007/s00330-022-09256-7

**Published:** 2022-11-17

**Authors:** Konstanze V. Guggenberger, Marius L. Vogt, Jae W. Song, Andreas M. Weng, Matthias Fröhlich, Marc Schmalzing, Nils Venhoff, Jost Hillenkamp, Mirko Pham, Stephan Meckel, Thorsten A. Bley

**Affiliations:** 1 Department of Diagnostic and Interventional Radiology, University Hospital Wuerzburg, Oberduerrbacher Straße 6, 97080 Wuerzburg, Germany; 2 Department of Diagnostic and Interventional Neuroradiology, University Hospital Wuerzburg, Wuerzburg, Germany; 3 Department of Radiology, Division of Neuroradiology, University of Pennsylvania, 3400 Spruce Street, Philadelphia, PA 19104 USA; 4 Department of Internal Medicine II, Rheumatology and Clinical Immunology, University Hospital Wuerzburg, Wuerzburg, Germany; 5 Department of Rheumatology and Clinical Immunology, Medical Center, Faculty of Medicine, University of Freiburg, Freiburg, Germany; 6 Department of Ophthalmology, University Hospital Wuerzburg, Wuerzburg, Germany; 7 Department of Neuroradiology, Medical Center, Faculty of Medicine, University of Freiburg, Freiburg, Germany; 8 RKH Klinikum Ludwigsburg, Ludwigsburg, Germany

**Keywords:** Giant cell arteritis, Magnetic resonance imaging, Orbit, Ophthalmic artery, Optic nerve

## Abstract

**Objective:**

Blindness is a feared complication of giant cell arteritis (GCA). However, the spectrum of pathologic orbital imaging findings on magnetic resonance imaging (MRI) in GCA is not well understood. In this study, we assess inflammatory changes of intraorbital structures on black blood MRI (BB-MRI) in patients with GCA compared to age-matched controls.

**Methods:**

In this multicenter case-control study, 106 subjects underwent BB-MRI. Fifty-six patients with clinically or histologically diagnosed GCA and 50 age-matched controls without clinical or laboratory evidence of vasculitis were included. All individuals were imaged on a 3-T MR scanner with a post-contrast compressed-sensing (CS) T1-weighted sampling perfection with application-optimized contrasts using different flip angle evolution (SPACE) BB-MRI sequence. Imaging results were correlated with available clinical symptoms.

**Results:**

Eighteen of 56 GCA patients (32%) showed inflammatory changes of at least one of the intraorbital structures. The most common finding was enhancement of at least one of the optic nerve sheaths (*N* = 13, 72%). Vessel wall enhancement of the ophthalmic artery was unilateral in 8 and bilateral in 3 patients. Enhancement of the optic nerve was observed in one patient. There was no significant correlation between imaging features of inflammation and clinically reported orbital symptoms (*p* = 0.10). None of the age-matched control patients showed any inflammatory changes of intraorbital structures.

**Conclusions:**

BB-MRI revealed inflammatory findings in the orbits in up to 32% of patients with GCA. Optic nerve sheath enhancement was the most common intraorbital inflammatory change on BB-MRI. MRI findings were independent of clinically reported orbital symptoms.

**Key Points:**

*• Up to 32% of GCA patients shows signs of inflammation of intraorbital structures on BB-MRI.*

*• Enhancement of the optic nerve sheath is the most common intraorbital finding in GCA patients on BB-MRI.*

*• Features of inflammation of intraorbital structures are independent of clinically reported symptoms.*

**Supplementary Information:**

The online version contains supplementary material available at 10.1007/s00330-022-09256-7.

## Introduction

Giant cell arteritis (GCA) is a challenging diagnosis due to a wide spectrum of nonspecific clinical symptoms. The most feared complication of GCA is vision loss due to inflammation of the posterior ciliary arteries, a branch of the ophthalmic artery, and is considered an ophthalmologic emergency. The reported rate of both visual complications and permanent vision loss in GCA ranges from 10 to 50% [1–4]. The posterior ciliary artery circulation is the primary source of blood supply to the anterior optic nerve head and to the choroidea. The central retinal artery supplies the retina. In GCA, arteritic anterior ischemic optic neuropathy is the result of inflammation of these respective ophthalmic branches which can result in permanent blindness [5]. Therefore, a prompt and accurate diagnosis to initiate therapy is critical to prevent vision loss.

In addition to the ophthalmic arteries and its branches [6], inflammation of other orbital structures has also been reported in GCA. MRI findings of inflammation of the optic nerve sheath [7–10], optic nerve [11], optic chiasm [9], and intraconal fat [7] and of the 3rd cranial nerve [12] have been reported. The spectrum of different anatomic patterns of orbital inflammation in GCA is not well understood and can be challenging to interpret diagnostically, as the imaging appearance can also mimic different inflammatory or infectious etiologies, such as idiopathic orbital inflammatory syndrome [7, 8, 13–15]. In this study, we investigated the prevalence and spectrum of orbital inflammatory findings in GCA patients who were imaged with postcontrast black blood MRI (BB-MRI) and compared the imaging findings to an age-matched control group.

## Methods

### Subjects

Following IRB approval for the study and after written informed consent, 56 consecutive patients with GCA and imaged with postcontrast BB-MRI between January 2019 and September 2020. These patients were retrospectively identified from a prospectively collected dataset from the University Hospitals Würzburg and Freiburg. Inclusion criteria were clinical or histologic diagnosis of GCA by temporal artery biopsy, age ≥ 50 years, and imaged with BB-MRI. A clinical diagnosis was established by a rheumatologist or ophthalmologist based on clinical criteria and the American College of Rheumatology 1990 criteria for the classification of GCA [16] without the use of BB-MRI. Fifty age-matched control cases from the University Hospital of Würzburg were also retrospectively identified from a central nervous system (CNS) cancer surveillance registry. The inclusion criteria for age-matched control cases were age ≥ 50 years, imaged with postcontrast BB-MRI, no clinical or laboratory diagnosis of vasculitis, no history of steroid therapy or other immunosuppressive therapy at time of the BB-MRI, and no evidence of CNS metastases or neoplasm on imaging.

### Imaging protocol

BB-MRI was performed on a 3-T scanner (MAGNETOM Prisma, Siemens Healthineers) using a dedicated 64-channel head coil. A whole-brain postcontrast compressed-sensing (CS)-accelerated, high-resolution black-blood 3D T1-weighted sampling perfection with application optimized contrasts using different flip angle evolution (SPACE) sequence optimized for intracranial vessel wall MR imaging was acquired in the sagittal plane [17]. To avoid motion artifacts, patients were instructed to close their eyes during the examination and to avoid eye movements as much as possible. Sequence parameters include isotropic spatial resolution 0.55 mm, TR/TE 800/10 ms, FOV 210 × 210 × 140 mm^3^, matrix 384 × 384 × 256^3^, pixel-bandwidth = 450 Hz/px, acquisition time 5:50 min. The sequence was performed 5 min after injection of a gadolinium-containing contrast agent (Dotagraf®, 0,5 mmol/ml).

### Image analysis

To conduct the study rigorously, all imaging data of both the GCA cohort and control cases were anonymized and uploaded into a folder. Two readers with > 3 years of dedicated neuroradiology training independently reviewed all BB-MRIs blinded to both clinical history/presentation and final diagnosis. Imaging endpoints included assessing for enhancement of the intraconal fat, the extraocular muscles, optic nerve sheath, optic nerve, optic chiasm, and vessel walls of the ophthalmic arteries for each orbit. Postcontrast enhancement of these structures was qualitatively assessed compared to adjacent nonenhancing soft tissue and considered a manifestation of inflammation when present. Inflammation of the optic nerve sheath was scored positive when both uniform concentric vessel wall thickening and enhancement was present in line with previously described findings [9]. Inflammation of the ophthalmic artery was considered when both concentric vessel wall thickening and contrast enhancement were present. Discordant results were reviewed and consensus reached by agreement between the raters.

### Statistical analysis

For statistical analysis, IBM SPSS Statistics (Version 28.0; IBM Corp) was used. Descriptive statistics were used. Continuous variables are presented as means and standard deviations and categorical variables as percentages. Percent agreement was calculated as the proportion of agreement between the raters. The association of presence of inflammatory intraorbital MRI findings with ophthalmologic symptoms was tested by chi-square analysis. A *p*-value of < 0.05 was considered to be statistically significant.

## Results

One-hundred and six subjects (58 female; mean age 72 years, SD 9) were included in the case-control study: 56 consecutive GCA patients (41 female; mean age 74 (range 61–89, SD 8) years) and 50 consecutive age-matched patients (17 female; mean age 70 (range 50–89, SD 10) years). At the time of BB-MRI, 20 patients were steroid-naïve and 36 patients received a mean of 70 days (range 1–560 (SD 133) days) of treatment. In all GCA patients, clinical diagnosis was established by a rheumatologist or ophthalmologist based on clinical criteria and the American College of Rheumatology 1990 criteria for the classification of GCA [16]. In 16 GCA patients, diagnosis was also confirmed by histology. Table 1 summarizes patients’ characteristics. Agreement between the two readers for all orbital image scoring was high (96–100%) for both the cases and control cohorts (Supplemental Table 1).

**Table 1:** Characteristics of total cohort, GCA cohort and control cohort

	Total cohort (*n* = 106)	GCA cohort (*n* = 56)	Control cohort (*n* = 50)
Mean age [years] (SD)	72 (SD 9)	74 (SD 8)	70 (SD 10)
Female [*n*]	58	41	17
Clinically diagnosed GCA	40	40	0
Histologically diagnosed GCA	16	16	0
Steroid-naive patients [*n*]	70	20	50

**Fig. 1:**
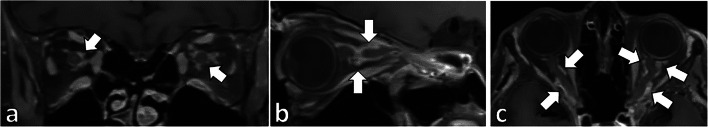
Bilateral optic nerve sheath enhancement in a patient diagnosed with GCA. (**a**) Coronal, (**b**) sagittal, and (**c**) axial postcontrast BB-MRI images show concentric thickening and contrast enhancement of the bilateral optic nerve sheaths (**a**–**c**, arrows) in a patient with GCA

**Fig. 2:**
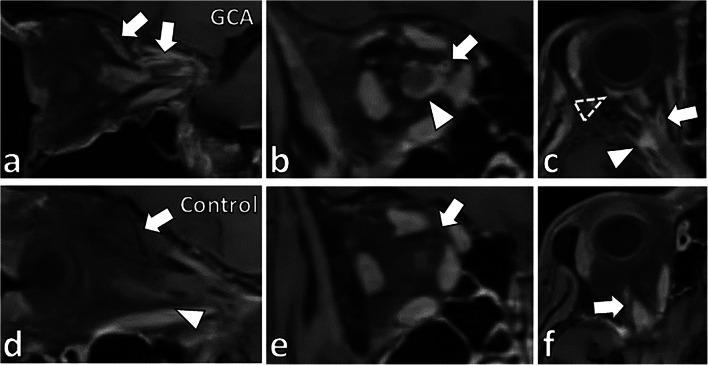
Vessel wall enhancement of the ophthalmic artery and its branches in a patient diagnosed with GCA. In a patient diagnosed with GCA, (**a**) sagittal, (**b**) coronal, and (**c**) axial BB-MRI images show concentric vessel wall thickening and enhancement of the right ophthalmic artery (**a**–**c**, arrows). There was also optic nerve sheath enhancement (**b**–**c**, arrowheads) and partial rim enhancement posterior to the globe (**c**, dashed arrowhead). In an age-matched control case, (**d**) sagittal, (**e**) coronal, and (**f**) axial BB-MRI show no vessel wall thickening or enhancement of the ophthalmic artery (**d**–**f**, arrows). No pathologic enhancement of the optic nerve sheath (**d**, arrowhead) or other orbital structures was identified

**Fig. 3:**
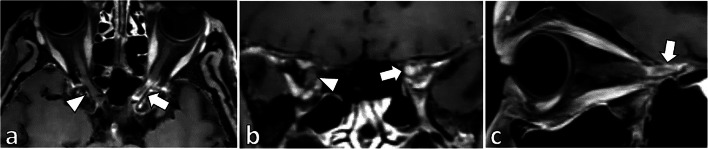
Left pre-chiasmatic optic nerve enhancement in a patient diagnosed with GCA. (**a**) Axial, (**b**) coronal, and (**c**) sagittal postcontrast BB-MRI images show contrast enhancement and inflammation of the left pre-chiasmatic optic nerve on BB-MRI (**a**–**c**, arrows), which contrasts with the normal right pre-chiasmatic optic nerve (**a** and **b**, arrowheads)

Among the 56 patients diagnosed with GCA, 18 (32%) showed inflammation of at least one of the orbital structures (Table 2). Inflammation of the optic nerve sheath was present in at least one orbit in 13 of 18 patients (72%). In 12 patients, the optic nerve sheath showed pathologic enhancement in both orbits (Fig. 1). In 8 patients (14%), the ophthalmic artery vessel wall showed inflammation in one orbit. Three patients (5%) showed bilateral ophthalmic artery vessel wall inflammation (Fig. 2). Three patients showed unilateral enhancement of the intraconal fat. One patient showed contrast enhancement of the intraorbital segment of the optic nerve (Fig. 3). Neither optic chiasm nor extraocular muscles exhibited any changes suspicious of inflammation in the GCA patients on BB-MRI. Table 2 summarizes the prevalence of inflammation of orbital structures in GCA patients on BB-MRI.

**Table 2:** Prevalence of inflammation of orbital structures on black blood MRI in patients with GCA

Orbital structure affected	Number (%) of GCA patients (total *n* = 56)
Intraconal fat	3 (5%) unilateral
	0 bilateral
Extraocular muscles	0
Optic nerve sheath	1 (2%) unilateral
	12 (21%) bilateral
Optic nerve	1 (2%), 0 bilateral
Optic chiasm	0
Ophthalmic artery vessel wall	5 (9%) unilateral
	3 (5%) bilateral

In 18 GCA patients, signs of inflammation were found on MR imaging. Only 7 of these 18 patients (7/18 = 39%) reported orbital symptoms at the time of MR imaging, ranging from pain to visual disturbances. The remaining 11 patients (11/18 = 61%) with signs of inflammation of intraorbital structures on BB-MRI were clinically asymptomatic. Additionally, only 14 of the 56 GCA patients in this study (25%) reported any ophthalmologic symptoms (Table 2). In these 14 patients, only 7 (50%) showed signs of inflammation on MR imaging. A subgroup analysis of these 14 GCA patients presenting with ophthalmologic symptoms was performed to examine prevalence of inflammatory intraorbital findings (Table 3). The prevalence of any positive intraorbital MR finding in the GCA group with ophthalmologic symptoms was 50% (*N* = 7 of 14) compared to 43% (*N* = 18 of 42) in the GCA group without ophthalmologic symptoms. Optic nerve sheath enhancement was the most common imaging finding in both subgroups with and without ophthalmic symptoms with up to 43% (*N* = 6) of GCA patients with ophthalmologic symptoms showing this finding compared to 17% (*N* = 7) in GCA patients without ophthalmologic symptoms. Additionally, ophthalmic artery vessel wall enhancement was also a frequent finding in GCA patients without ophthalmologic symptoms with 17% (*N* = 7) showing unilateral or bilateral findings. Despite the absence of ophthalmologic symptoms, 11 of 42 GCA patients (26%) showed signs of inflammation of any of the analyzed intraorbital soft tissue structures on BB-MRI. There was no significant association between positive intraorbital MR findings and ophthalmologic symptoms among patients diagnosed with GCA (*p* = 0.10).

**Table 3:** Prevalence of contrast enhancement of specific orbital structures on MRI in GCA patients with and without ophthalmologic symptoms

Orbital structure affected	Number (%) of GCA patients with ophthalmologic symptoms (*n* = 14)	Number (%) of GCA patients without ophthalmologic symptoms (*n* = 42)
Intraconal fat	0	3 (7%) unilateral
		0 bilateral
Extraocular muscles	0	0
Optic nerve sheath	0 unilateral	1 (2%) unilateral
	6 (43%) bilateral	6 (14%) bilateral
Optic nerve	0	1 (2%), 0 bilateral
Optic chiasm	0	0
Ophthalmic artery vessel wall	0 unilateral	5 (12%) unilateral
	1 (7%) bilateral	2 (5%) bilateral

Table 3 shows the prevalence of contrast enhancement of specific orbital structures on MRI in a subgroup analysis in GCA patients with and without ophthalmologic symptoms.

There was also no correlation between positive intraorbital MR findings and vessel wall enhancement of the intracranial arteries. Only one patient of the GCA cohort showed unilateral long-segment enhancement and thickening of the vessel wall of the right internal carotid artery and of the right middle cerebral artery. However, this patient did not show vessel wall enhancement of the ophthalmic artery or any other abnormalities of intraorbital soft tissue structures.

No pathologic orbital enhancement or false-positive findings were present to suggest inflammation in any of the age-matched control cases.

## Discussion

The results of this study show inflammation manifested by contrast enhancement of the optic nerve sheath can be seen in up to 23% of patients diagnosed with GCA with or without ophthalmologic symptoms. In fact, 17% of GCA patients without clinical ophthalmologic signs or symptoms also presented with optic nerve sheath enhancement. Contrast enhancement of the intraconal fat was the next most prevalent imaging finding in these patients. By contrast, in the age-matched control group, none showed contrast enhancement of any orbital structures. Interestingly, in a subgroup analysis of patients without ophthalmologic signs and symptoms, 17% revealed contrast enhancement of the optic nerve sheath and ophthalmic artery vessel walls. This finding was surprising given the absence of ophthalmic clinical signs and symptoms and highlight the potential role of neuroimaging in GCA patients for detecting subclinical inflammation and disease in orbital structures.

Recent changes to the EULAR guidelines recommend a role for neuroimaging in GCA patients in diagnosis [18]. However, the recommendations are based on evaluating vessel wall enhancement of the temporal arteries of the scalp and little is understood about the role of neuroimaging for orbital involvement. Several studies have indeed reported high sensitivity and specificity for diagnosing GCA on BB-MRI due to the ability to detect vessel wall inflammation of the scalp arteries (temporal and occipital arteries) [19]. BB-MRI has also been shown to detect vessel wall enhancement of the ophthalmic arteries with a reported sensitivity and specificity of 100% [20]. In this study, although the pulse sequence was not designed to evaluate the orbital structures, our results show that pathologic orbital findings can still be detected on intracranial BB-MRI and may provide important diagnostic information in patients being evaluated for GCA. Given the ability to comprehensively evaluate the full length of many affected arteries in GCA, BB-MRI may be further able to differentiate subgroups of GCA, specifically cranial (only scalp artery involvement) GCA and ocular GCA (scalp and orbital involvement or isolated orbital involvement).

There is a wide range of possible BB-MRI pulse sequences that can be used to visualize vessel wall enhancement of the scalp arteries as well as orbital structures [21]. An active area of technical development for BB-MR imaging is to optimize pulse sequences for the anatomy of interest. However, there are several challenges in orbital MR imaging, which include susceptibility artifacts due to the air/bone interface of the orbits and paranasal sinuses, which often result in poor fat suppression, as well as motion degradation due to movement of the globe during long acquisition times. Sensitivity for detecting contrast enhancement of orbital structures due to these imaging pitfalls may be lower compared to dedicated orbital MR imaging with additional pulse sequences, such as STIR imaging or fat suppressed T1-weighted postcontrast images with Dixon technique. The selection of the optimal pulse sequence for evaluating the orbits in suspected GCA patients has not been fully investigated and is a future direction. Notably, in the absence of clinical ophthalmologic symptoms in patients with suspected GCA, ordering an additional dedicated orbital MRI is likely not cost-effective for patients and hospitals. As a screening MR exam, if the orbits are included in scalp or intracranial BB-MRI imaging for temporal artery assessment, it is prudent for the radiologist to include orbital assessment and be aware of these possible imaging findings during image interpretation. To detect vessel wall enhancement of the ophthalmic artery, confirming arterial anatomy and distinguishing artery from the superior ophthalmic vein is important. In the absence of a magnetic resonance angiography exam, following the ophthalmic artery from the origin at the internal carotid artery can be helpful.

Our study also revealed an overall lower prevalence than other studies in inflammatory orbital findings on MRI compared to previously published studies. For example, Geiger et al reported vessel wall contrast enhancement of the ophthalmic arteries in 46% of GCA patients on axial post-contrast T1-weighted spin-echo imaging [6] compared to 14% in our study. This higher prevalence could be explained by differences in imaging technique as Geiger et al performed imaging at higher spatial resolutions. In our subgroup analysis, patients presenting with ophthalmologic symptoms also showed a higher prevalence of contrast enhancement of orbital structures. However, the small sample size of this subgroup is a limitation of this study and a larger sample size may be needed to confirm this observation. Moreover, in our cases, 20 of the 56 GCA patients were steroid-naïve at time of MR imaging. The remaining 35 GCA patients had received steroids for a mean of 70 days (range 1–560 days) prior to MR imaging. Studies suggest the sensitivity of MRI for detecting vasculitis decreases significantly within 5 days after therapy initiation [19], and this may also lead to lower prevalence rates of orbital contrast enhancement. A future direction is to examine how steroid treatment and the duration of treatment affects contrast enhancement of orbital structures. However, notably, the age-matched control group did not exhibit any signs of inflammation of orbital structures, which strengthens the observation in our analyses. The lack of false-positives likely results from the fact that these control cases were originally imaged for brain tumor/metastases surveillance and showed no clinical or laboratory evidence of vasculitis.

Another limitation of this study is the relative paucity of ophthalmologic examination details. Descriptions of ocular symptoms and ophthalmologic examination findings, such as specification of the affected side, would have allowed for a more specific correlation of clinical and imaging findings. The retrospective nature of this study limited such detailed information from the patient’s clinical history. Finally, histologic confirmation of inflammation of the contrast enhancing orbital structures was not performed and would not have been ethically justifiable. There are case reports with histologic confirmation of inflammation and occlusion of the posterior ciliary arteries [22] and radiology-pathology correlation studies showing presence of giant cells and granulomatous inflammation of the optic nerve sheath and extraocular muscles in patients with GCA [7, 8]. The reported MR imaging findings from these case reports with histologic confirmation are comparable to our data [8].

## Conclusions

Independent of ophthalmologic symptoms, 32% of patients diagnosed with GCA patients showed MRI signs of inflammation of intraorbital structures. Contrast enhancement of the optic nerve sheath was the most prevalent imaging finding. High-resolution compressed-sensing (CS) SPACE T1 black blood MRI may be a sensitive noninvasive imaging technique to detect intraorbital manifestations in cranial GCA and have a diagnostic role in detecting ophthalmologic complications.

## Supplementary Information

ESM 1 (DOCX 35 kb)

## Abbreviations

BB-MRI: Black blood MRI
CNS: Central nervous system
CS: Compressed-sensing
GCA: Giant cell arteritis
MRI: Magnetic resonance imaging
SPACE: Sampling perfection with application optimized contrasts using different flip angle evolution

## Declarations

### Guarantor

The scientific guarantor of this publication is Prof. Dr. Thorsten Alexander Bley.

### Statistics and biometry

Dr. Andreas Max Weng (one of the Co-authors) kindly provided statistical advice for this manuscript.

### Methodology

• prospective

• case-control study

• bicentric study
