# Supplementary material for: Intraorbital findings in giant cell arteritis on black blood MRI
Source: Eur Radiol. 2022 Nov 17;33(4):2529–35. doi: 10.1007/s00330-022-09256-7 (PMC10017783; doi:10.1007/s00330-022-09256-7)
Supplement: Supplementary file 1 — (DOCX 35 kb) [file 330_2022_9256_MOESM1_ESM.docx]

**Supplemental Table 1**: concordance rates of MR image analysis between the two readers for each of the analyzed intraorbital structures for the total cohort and the GCA collective.

|  | GCA patients (n=56) | | | | | | | | | | | |
| --- | --- | --- | --- | --- | --- | --- | --- | --- | --- | --- | --- | --- |
|  | **intraorbital fat** | | **extraocular muscles** | | **optic nerve** | | **optic nerve sheath** | | **optic chiasm** | | **ophthalmic artery** | |
|  | right | left | right | left | right | left | right | left | right | left | right | left |
| **absolute** | 56 | 55 | 56 | 56 | 56 | 56 | 56 | 55 | 56 | 56 | 55 | 54 |
| **Percentage (%)** | 100 | 98 | 100 | 100 | 100 | 100 | 100 | 98 | 100 | 100 | 98 | 96 |
|  | age matched control cohort (n=50) | | | | | | | | | | | |
|  | **intraorbital fat** | | **extraocular muscles** | | **optic nerve** | | **optic nerve sheath** | | **optic chiasm** | | **ophthalmic artery** | |
|  | right | left | right | left | right | left | right | left | right | left | right | left |
| **absolute** | 50 | 50 | 50 | 50 | 50 | 50 | 50 | 50 | 50 | 50 | 50 | 50 |
| **percentage (%)** | 100 | 100 | 100 | 100 | 100 | 100 | 100 | 100 | 100 | 100 | 100 | 100 |
